# Reduction of Nitroaromatics by Gold Nanoparticles on Porous Silicon Fabricated Using Metal-Assisted Chemical Etching

**DOI:** 10.3390/nano13111805

**Published:** 2023-06-05

**Authors:** Ling-Yi Liang, Yu-Han Kung, Vincent K. S. Hsiao, Chih-Chien Chu

**Affiliations:** 1 Department of Medical Applied Chemistry, Chung Shan Medical University, Taichung 40201, Taiwan; s0964026@csmu.edu.tw; 2 Department of Applied Materials and Optoelectronic Engineering, National Chi Nan University, Nantou 54561, Taiwan; s108328040@ncnu.edu.tw; 3 Department of Medical Education, Chung Shan Medical University Hospital, Taichung 40201, Taiwan

**Keywords:** porous silicon, metal-assisted chemical etching, gold nanoparticle, catalyst, nitroaromatic, *p*-nitroaniline

## Abstract

In this study, we investigated the use of porous silicon (PSi) fabricated using metal-assisted chemical etching (MACE) as a substrate for the deposition of Au nanoparticles (NPs) for the reduction of nitroaromatic compounds. PSi provides a high surface area for the deposition of Au NPs, and MACE allows for the fabrication of a well-defined porous structure in a single step. We used the reduction of *p*-nitroaniline as a model reaction to evaluate the catalytic activity of Au NPs on PSi. The results indicate that the Au NPs on the PSi exhibited excellent catalytic activity, which was affected by the etching time. Overall, our results highlighted the potential of PSi fabricated using MACE as a substrate for the deposition of metal NPs for catalytic applications.

## 1. Introduction

Noble metal nanoparticles (NPs) have attracted considerable interest because of their unique chemical and physical properties, including their electronic [1], optical [2], magnetic [3], and catalytic properties [4]. These properties distinguish noble metal NPs from bulk metals and make them highly useful for a wide range of applications, such as catalysis, sensing, optics, and fuel cells. In various oxidation and reduction reactions, Au NPs have demonstrated great potential as highly efficient catalysts [5]. However, the tendency of NPs to aggregate in solutions because of their high surface energy may reduce their catalytic activity. To solve this problem, different approaches have been explored for stabilizing NPs, including capping them with organic molecules [6] or polymers [7] or dispersing them onto solid supports [8]. However, although organic molecules or polymers can serve as capping agents to prevent aggregation, they may also reduce the catalytic activity of NPs. By contrast, solid supports offer higher stability but often require time-consuming separation procedures to isolate the catalysts from the reaction system. Two-dimensional (2D) graphene, which has unique properties, such as a high specific surface area, excellent electrical conductivity, high charge carrier mobility, and high mechanical strength, has emerged as a promising support for various types of NPs [9]. Considerable interest has also been directed toward constructing size- and shape-controlled noble metal NPs supported on 2D carbon materials [10]. However, at the nanoscale, metal particles with highly active centers are not at thermodynamic equilibrium and are prone to aggregation with solid supports. Therefore, strategies for stabilizing NPs, such as capping them with multifarious stabilizers, designing core–shell structures, or anchoring them onto specific supports, must be explored.

Aromatic amines are crucial building blocks in organic synthesis and in the pharmaceutical industry [11]. Their synthesis can be achieved by reducing corresponding nitroaromatics [12]. However, these reduction reactions require catalysts for efficient conversion [13]. Although hydrogen gas and NaBH_4_ are commonly used as reducing agents, noble metal NPs, such as Pt, Au, Ag, and Pd NPs, have been reported to be effective catalysts in the presence of NaBH_4_ [14,15,16,17,18]. However, the aggregation of these NPs in the reaction system limits their catalytic efficiency, necessitating the development of novel techniques for ensuring well-dispersed stabilization of the NPs. To this end, various materials have been explored for stabilizing nanosized noble metal catalysts for catalyzing nitroaromatics. Kim et al. [19] synthesized a core–satellite structure using poly(N-isopropylacrylamide-acrylamide) and Au NPs for the photothermal-mediated catalytic reduction of 4-nitrophenol. Dong et al. [20] prepared Ag NPs dispersed in a nano-silica nanocatalyst, which exhibited excellent catalytic activity in the reduction of 4-nitrophenol and 2-nitroaniline using NaBH_4_ in water at room temperature. Importantly, the nanocatalyst could be easily recovered and reused for at least ten cycles in both reduction reactions, demonstrating its good stability. Pandey et al. [21] synthesized highly stable dispersions of Pt NPs in guar gum, a natural, non-toxic, and eco-friendly biopolymer, serving as both the reducing and capping agent precursor in an aqueous medium. The catalytic activity of biopolymer-supported Pt NPs was demonstrated in the liquid-phase reduction of p-nitrophenol and p-aminophenol. The catalytic reduction of nitroaromatics achieved a remarkable efficiency of 97% within a total reaction time of 320 s at room temperature. Graphene oxide (GO) and reduced graphene oxide (r-GO) were also utilized to stabilize the noble NPs [22,23]. Without the need for additional reductants, surfactants, or protecting ligands, metallic noble metals were deposited on partially r-GO mats through a simple redox reaction between noble metal precursors and GO in an aqueous solution. These GO- or r-GO-supported noble NPs exhibited excellent catalytic activity for the selective reduction of nitroaromatic compounds. Cai et al. [24] developed a novel nanostructured catalyst comprising small and uniform Au NPs with a diameter of approximately 5 nm and ceria nanotubes (CeO_2_ NTs). The catalytic performance of the Au NPs/CeO_2_ NT catalyst in the reduction of 4-nitrophenol to 4-aminophenol was significantly higher compared to similar catalysts composed of chemically prepared AuNPs or commercially available CeO_2_ powder as the support. The superior catalytic activity can be attributed to the unique surface properties of the synthesized Au NPs/CeO_2_ NT catalyst, as well as the interaction between the barrier-free surface of Au NPs and surface defects (oxygen vacancies) of CeO_2_ NTs, leading to the presence of oxidized Au species. Chen et al. [25] conducted a comprehensive investigation of the reduction of p-nitrophenol by NaBH_4_ in the presence of raspberry-like composite sub-microspheres composed of poly(allylamine hydrochloride)-modified polymer poly(glycidyl methacrylate) with tunable Au NPs. They systematically examined the effects of polyelectrolyte concentration, the ratio of polymer spheres to Au NPs, and solution pH during composite synthesis on various reaction parameters such as the induction period, reaction time, average reaction rate, and average turnover frequency. They also proposed a mechanism to explain the observed enhancement in catalytic activity, which involves the active epoxy groups present on the polymer spheres and the strong adsorption of p-nitrophenolate anions onto the positively charged spheres.

Metal-assisted chemical etching (MACE) is a simple and versatile method for fabricating porous silicon (PSi) structures without requiring electrochemically etched electrodes [26]. The main principle of MACE is to deposit a noble metal on the surface of a Si substrate and then immerse the substrate in an etching solution containing fluoride and an oxidizing agent to induce an etching reaction [27]. MACE can be used to produce various PSi structures for which the pore size, porosity, and surface morphology can be controlled by adjusting the composition and concentration of the etching solution as well as the type, thickness, and distribution of the metal catalyst [28]. In conventional MACE, a mixture of HF and H_2_O_2_ is commonly used as an etching solution [29]. HF reacts with the oxygen atoms on the surface of the Si substrate to form fluorosilicic acid, which further dissolves the Si surface. Simultaneously, the noble metal catalyst on the surface of the Si substrate serves as an active site for catalyzing the etching reaction by promoting the generation of holes (positive charges) in the Si substrate through the reduction of H_2_O_2_. These holes are then injected into the interface between the Si substrate and the metal catalyst, resulting in the oxidation and dissolution of the Si substrate in the etching solution [30]. During the etching process, the metal catalyst serves as a cathodic reaction zone, whereas the Si substrate serves as an anodic reaction zone [31].

Because of their high surface area and tunable pore size, PSi or PSi substrates have been widely used for the deposition of metal NPs for catalyzing nitroaromatic compounds [32,33,34] in a different fabrication technique. To our knowledge, this is the first study to explore the use of PSi fabricated using MACE as a substrate for the deposition of Au NPs for the reduction of nitroaromatic compounds. As a substrate, PSi provides a high surface area for the deposition of Au NPs, and MACE allows for the fabrication of a well-defined porous structure with a tunable pore size. In this study, we used scanning electron microscopy (SEM) and energy-dispersive X-ray spectroscopy (EDS) to characterize Au NPs on PSi. We used *p*-nitroaniline (PNA) reduction as a model reaction to evaluate the catalytic activity of Au NPs. Our results indicate that the Au NPs on PSi exhibited excellent catalytic activity toward the reduction of PNA. They also indicated that the catalytic activity of the PSi substrate was affected by the etching time. That is, as the etching time increased, the surface area of the PSi increased, which increased the atomic weight percentage of Au NPs immobilized on the surface. Because the surface area available for the catalytic reaction increased, the catalytic activity also increased. However, at a certain point, further increasing the etching time resulted in a decrease in catalytic activity, which may have been attributable to the aggregation of Au NPs. Overall, these findings may have major implications for the development of efficient and cost-effective catalysts for various organic transformations.

## 2. Materials and Methods

Briefly, N-type Si wafers with a resistivity of 1–10 Ω·cm and a crystal orientation of (100) were cut using a glass cutter into 1.5 × 1.5 cm^2^ square pieces, ultrasonically cleaned with methanol, acetone, and deionized (DI) water for 15 min in each solution, and dried with a nitrogen gas gun. The cleaned Si substrates were then placed vertically in an acid-resistant Teflon cell of 20 mL in size. Subsequently, a MACE mixture containing HF (48%), H_2_O_2_ (30%), DI water, and HAuCl_4_ (3 mM) at a ratio of 1:5:2:4 (volume ratio) of 12 mL was added to the Teflon cell. Etching was then conducted at room temperature without stirring for different durations. After the etching process was completed, the PSi substrate was removed from the etching solution and rinsed with anhydrous alcohol and DI water to remove any residual HF solution. Finally, the PSi substrate was dried using a nitrogen gas gun. The fabrication process of electrochemically etched PSi is described in detail in previous studies [35]. In detail, a ±20 V, 40 W source measure unit (PXI 4130) was used as power supply for offering a constant voltage mode at a current density of 30 mA/cm^2^ for 30 min. An etching solution containing HF, ethanol, and DI water at a ratio of 1:2:1 (volume ratio) of 12 mL was added. A catalytic solution containing 10 mL of PNA at various concentrations and 33 mg of NaBH_4_ was then premixed for 1 h using a magnetic stirrer. The prepared PSi and catalytic solution were then placed together in a glass vial for absorbance measurement at various time intervals. Finally, surface morphological analysis, elemental analysis, and EDS mapping were performed using a multifunction environmental field emission scanning electron microscope equipped with an energy-dispersive X-ray spectrometer (Hitachi SU-5000, Hitachi, Tokyo, Japan).

## 3. Results and Discussion

**Figure 1:**
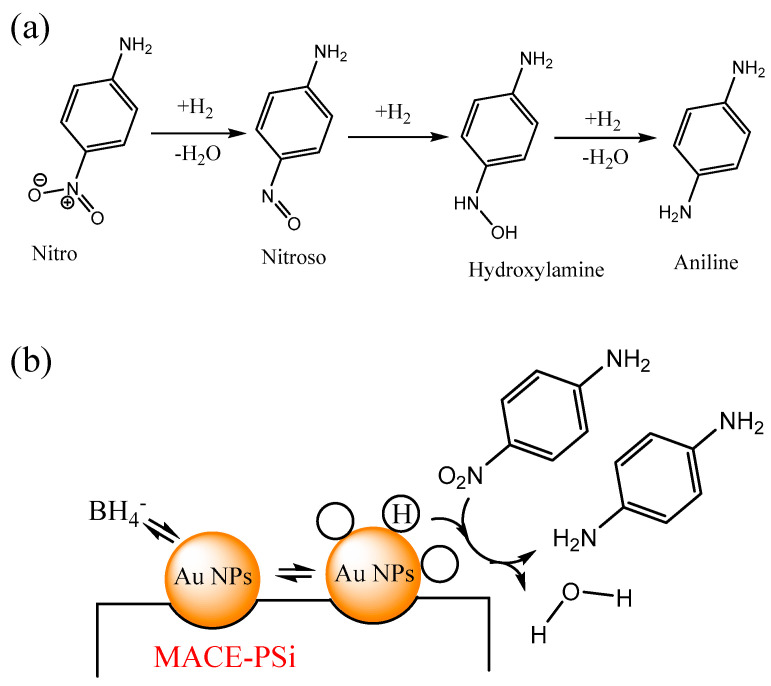
(**a**) Conversion of a nitroaromatic molecule to an aniline molecule. (**b**) Schematic of the catalytic reduction mechanism of PNA with Au NPs. Hydrogen and PNA adsorb on the surface of Au NPs, and the nitro group is reduced into a nitroso group. Further hydrogenation results in the formation of hydroxylamine, which undergoes dehydration to produce the final product, PPD.

The conversion of a nitroaromatic molecule to an aniline molecule involves the hydrogenation–dehydration of nitroaniline to form a nitrogen–oxygen double bond, followed by hydrogenation to produce hydroxylamine, and finally, further hydrogenation–dehydration to yield the product, p-phenylenediamine, as shown in Figure 1a. The hydrogenation–dehydration of nitroaniline: Nitroaniline undergoes hydrogenation–dehydration under appropriate conditions and in the presence of a suitable catalyst such as nickel or platinum. This reaction leads to the removal of the nitro group (NO_2_) and the formation of a nitrogen–oxygen double bond. In the compound formed in the previous step, the nitrogen–oxygen double bond reacts with hydrogen gas, resulting in the formation of hydroxylamine (NH_2_OH). This step involves a hydrogenation reaction where the nitrogen–oxygen double bond is reduced to an amino group. The final step involves the hydrogenation–dehydration of hydroxylamine. Under suitable conditions, hydroxylamine reacts with hydrogen gas once again, undergoing dehydration. This reaction removes the hydroxyl group (OH) and ultimately yields p-phenylenediamine.

Figure 1b depicts the reduction of PNA to *p*-phenylenediamine (PPD) in the presence of NaBH_4_ through the catalytic reaction of Au NPs inside PSi fabricated using MACE. The mechanism of nitroaromatic reduction catalyzed by Au NPs involves four steps: adsorption, hydrogen atom generation, activation, and product formation. In the first step, a nitroaromatic molecule adsorbs, either chemically or physically, onto the Au surface, where it interacts with the surface electrons of Au NPs. In the second step, hydrogen gas molecules adsorb on the Au surface and dissociate into hydrogen atoms. This dissociation reaction is promoted by Au, which acts as a catalyst. In the third step, the nitroaromatic molecule is activated by the adsorbed hydrogen atoms, and the nitro group is reduced to an amino group, forming an intermediate aminoaromatic compound. In the fourth step, the intermediate aminoaromatic compound undergoes further reaction to form a corresponding reduced product. This product desorbs from the Au surface through different pathways.

**Figure 2:**
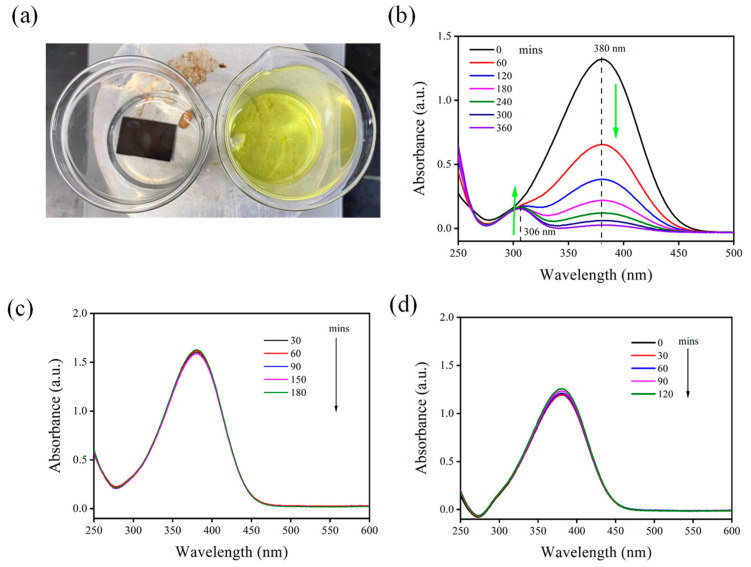
(**a**) PNA with (left) and without (right) PSi with the solution kept at room temperature for 60 min. Absorption spectra of PNA (**b**) with MACE-PSi, (**c**) without MACE-PSi, and (**d**) with electrochemically etched PSi.

In the presence of NaBH_4_, the reaction between PNA and PPD was detected by monitoring the absorption spectra of the solution. In this reaction, the N–H bond of nitroaniline underwent a hydrogen transfer process with the hydrogen atoms of NaBH_4_, resulting in the formation of PPD. Over time, the reduction of PNA lowered the degree of absorption, and a new absorption peak corresponding to PPD emerged. Consequently, the color of the solution changed from yellow, indicating the presence of PNA, to transparent, indicating the presence of PPD. This reaction was visible to the naked eye. As shown in Figure 2a, the beakers on the right and left contain identical mixtures of PNA and NaBH_4_. However, the beaker on the left contained MACE-PSi, whereas that on the right did not. After the mixture was allowed to stand for 1 h, the difference in color between the two solutions became evident to the naked eye. Specifically, the solution on the left, which contained PSi, changed from yellow to transparent, whereas the solution on the right, which lacked PSi, remained unchanged. During the catalytic reaction, absorption spectra were used to monitor the reduction of PNA to PPD (Figure 2b). In the ultraviolet–visible spectral region, the peak position of PNA was located between 300 and 400 nm, which differed from that of PPD. Therefore, whether a reaction occurred was determined by comparing the absorption spectra before and after the reaction. As the reaction proceeded, the characteristic peak of PNA at approximately 400 nm gradually decreased, whereas the peak of PPD at approximately 300 nm gradually increased. The disappearance of a peak at 400 nm and the appearance of a peak at 300 nm verified the successful reduction of PNA to PPD. As presented in Figure 2c, no changes in absorption were observed in a controlled experiment without the addition of PSi. The absorption spectra of the PNA/NaBH_4_ solution remained unchanged, confirming the catalytic function of MACE-PSi. Therefore, electrochemically etched PSi was used as a catalytic substrate, and the same catalytic experiments were conducted. In addition, temporal changes in solution absorption were recorded. As indicated in Figure 2d, because PSi contains no metallic Au, the absorbance of the solution remained unchanged.

**Figure 3:**
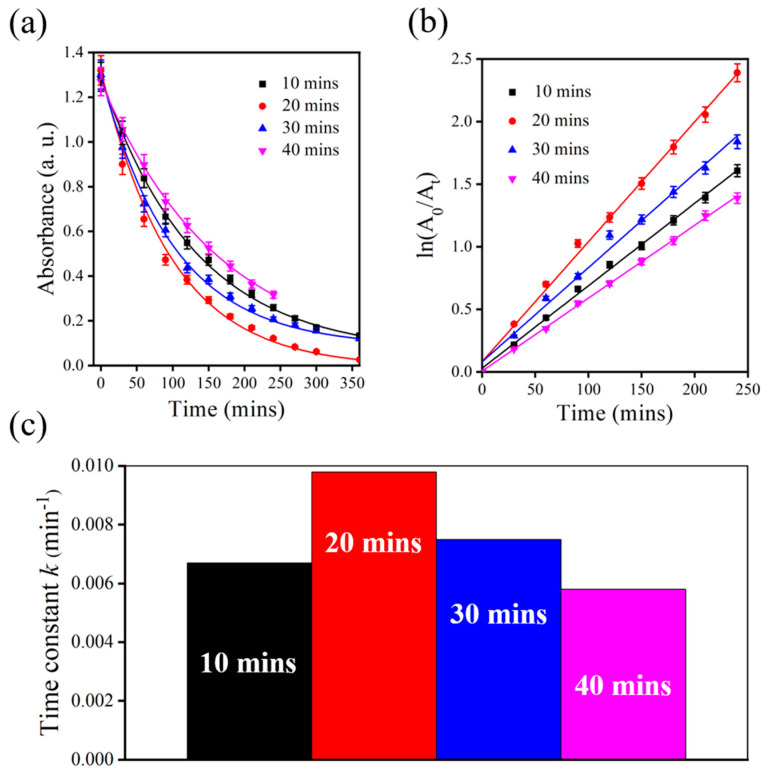
(**a**) Peak absorption (380 nm) changes in PNA with PSi over various etching times. (**b**) Calculated absorption values over time with MACE-PSi as a catalyst. (**c**) Calculated time constant values with PSi over various etching times.

Generally, the etching time of PSi is the most direct experimental parameter for modifying the surface morphology of PSi. Studies have indicated that the surface morphology of PSi can be used to determine changes in the deposition of Au thin films in surface-enhanced Raman scattering [35]. In the current study, PSi was fabricated over various etching times, and its catalytic effects on PNA were compared under the same catalytic conditions. The correlation between etching time and catalytic efficiency was also investigated by analyzing changes in the absorbance spectrum of PNA at a peak wavelength of 380 nm. Figure 3a presents the time-dependent absorbance peaks of PNA at 380 nm that were observed in the presence of PSi over various etching times. All PSi substrates prepared over various etching times and served as catalyst materials for PNA. However, the sample etched for 20 min exhibited the highest catalytic performance. After the initial absorbance peak intensity (*A*_0_) was compared with the absorbance peak intensity after a certain period of time (*A_t_*), a logarithmic calculation was performed to establish the correlation between the calculated values and elapsed time (Figure 3b). According to the results, the PSi sample etched for 20 min exhibited the highest catalytic performance for PNA. Therefore, the reduction rate and time constant (*k*) values were calculated using a pseudo first-order reaction [14], as shown below:

(1) $$\ln(A_{0}/A_{t})=kt$$

The results are presented in Figure 3c. The PSi sample etched for 20 min exhibited the highest catalytic constant, followed by the sample etched for 30 min, whose catalytic constant was higher than that of the sample etched for 10 min but lower than that of the sample etched for 20 min. Of the samples, the PSi sample etched for 40 min exhibited the lowest catalytic performance for PNA.

Auric acid (HAuCl_4_) is utilized as a metal catalyst in the MACE process to fabricate PSi, similar to the use of silver nitrate [28]. Auric acid, a metal salt containing gold (Au), acts as a catalyst in the HF etching solution during the MACE process. The gold ions (Au^3+^) within auric acid react with silicon fluoride (SiF_6_^2−^) present in the etching solution. This reaction leads to the dissolution of Au species and the etching of the silicon material. By adjusting the concentration of auric acid, it is possible to control the formation of pores and the structural characteristics of porous silicon during the MACE process [29]. When auric acid is employed as the metal catalyst in MACE, the internal structure of PSi can contain gold nanoparticles [28]. This occurs due to the reduction of Au^3+^ from HAuCl_4_ during the etching process, resulting in their deposition inside the pores of the silicon material. When the silicon material is immersed in the etching solution containing HAuCl_4_, the Au^3+^ ions react with the silicon material present in the solution. Through this process, Au^3+^ ions are reduced to gold atoms, which then deposit inside the pores, forming gold nanoparticles within the PSi.

**Figure 4:**
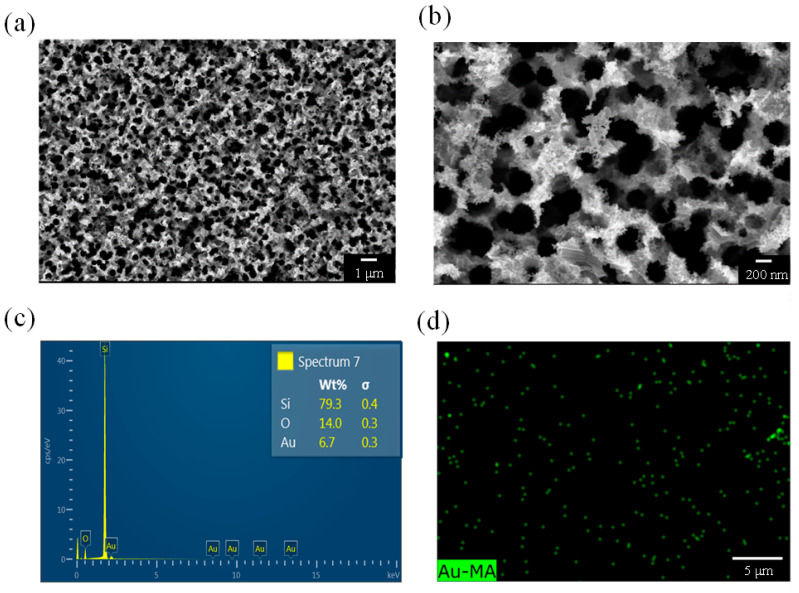
(**a**,**b**) SEM images, (**c**) EDS analysis, and (**d**) EDS mapping of a PSi sample etched for 10 min.

**Figure 5:**
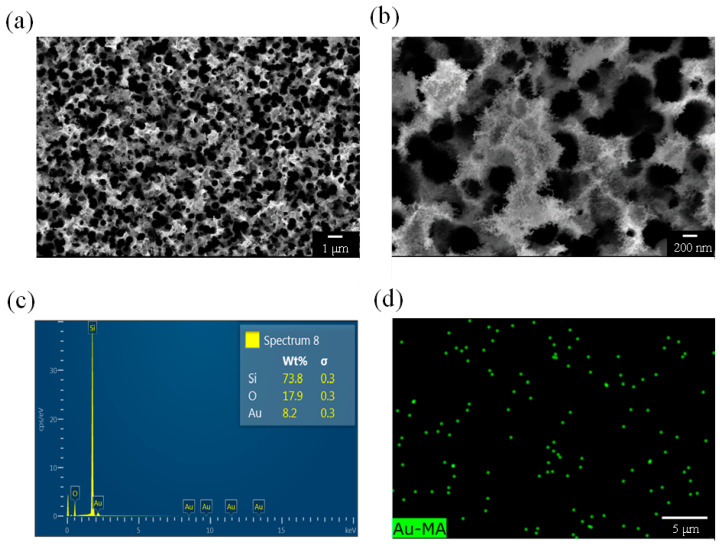
(**a**,**b**) SEM images, (**c**) EDS analysis, and (**d**) EDS mapping of a PSi sample etched for 20 min.

**Figure 6:**
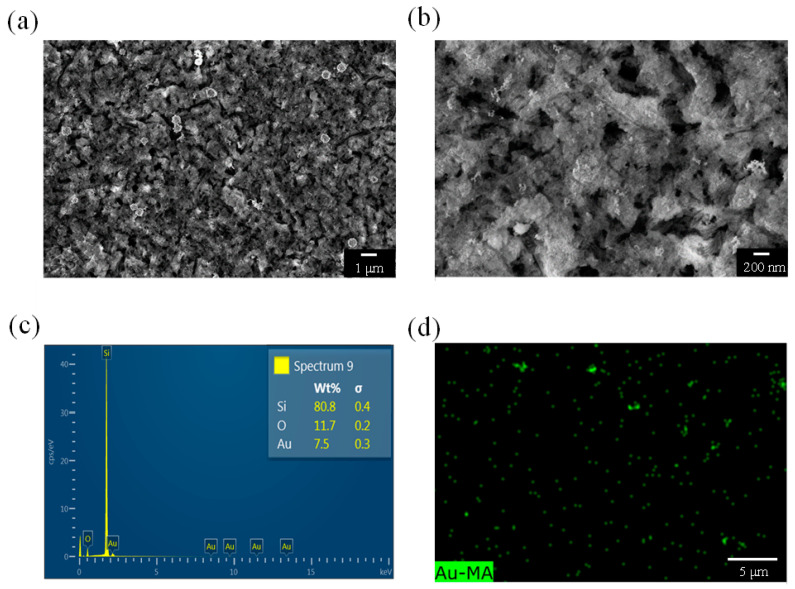
(**a**,**b**) SEM images, (**c**) EDS analysis, and (**d**) EDS mapping of a PSi sample etched for 30 min.

**Figure 7:**
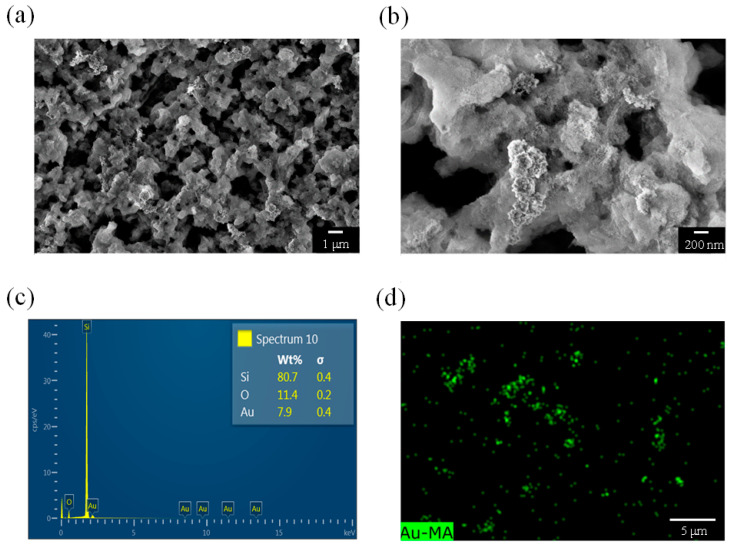
(**a**,**b**) SEM images, (**c**) EDS analysis, and (**d**) EDS mapping of a PSi sample etched for 40 min.

Figure 4, Figure 5, Figure 6 and Figure 7 present both the surface morphological and elemental analysis results for the PSi prepared over various etching times and the EDS mapping results for the Au elements. Figure 4a is an SEM image of a PSi after 10 min of etching and reveals a porous structure. Figure 4b is an SEM image of the same sample but at a higher magnification and reveals a pore diameter of approximately 250 nm and fluff-like protrusions resembling Au structures at the locations at which the pores connect to one another. As indicated in Figure 4c, elemental analysis verified the presence of Au. Figure 4d is an EDS mapping image generated using Au elements, with green dots indicating the locations of Au. Both surface morphological analysis and elemental analysis confirmed the existence of a porous structure and Au in the PSi sample.

Using the same analytical techniques, we analyzed a PSi sample etched for 20 min (Figure 5). As shown in Figure 5a, etching for 20 min resulted in larger pores in the PSi, indicating a smaller diameter of Si. As indicated in Figure 5b, the pore size of the PSi was approximately 300 nm, which is larger than that of the PSi sample etched for 10 min. The figure also clearly reveals fluffy protrusions on the surface of the PSi, which are distributed more uniformly than those on the surface of the PSi sample etched for 10 min (Figure 4b). Figure 5c depicts the elemental analysis results for the PSi sample etched for 20 min, with the results indicating a higher Au content than that of the PSi sample etched for 10 min. The distribution of Au being uniform and the Au content being high may indirectly explain why the PSi sample etched for 20 min had a stronger PNA catalytic effect than that of the PSi sample etched for 10 min. Figure 5d depicts the EDS mapping results of the Au elements, with green dots indicating the locations of Au. Although the number of green dots in the sample etched for 20 min was not larger than that of the green dots in the sample etched for 10 min, the sample etched for 20 min was clearly brighter, confirming a difference in the amount of Au and the presence of fluffy protrusions on the surface of the PSi sample etched for 20 min.

Figure 6a depicts the surface morphology of a PSi sample etched for 30 min. A comparison with the samples etched for 10 and 20 min indicated a decrease in the pore size of the PSi sample etched for 30 min. As shown in Figure 6b, the pore diameter was approximately 150 nm, and the diameter of the PSi substantially increased. However, the hairy protrusions observed in the samples that were etched for 10 and 20 min were not observed on the surface of the PSi sample that was etched for 30 min. In addition, elemental analysis revealed a weight percentage of 7.5 wt% for Au in the PSi sample etched for 30 min, which was higher than that of the PSi sample etched for 10 min (6.7 wt%) but lower than that of the PSi sample etched for 20 min (8.2 wt%). These findings may explain why the PSi sample etched for 30 min was more effective than the PSi sample etched for 10 min at catalyzing PNA but not as effective as the PSi sample etched for 20 min. As shown in Figure 6d, Au element mapping revealed an uneven distribution of Au on the surface of the PSi, with some green dots exhibiting aggregation.

Figure 7 depicts the SEM and EDS results of a PSi sample etched for 40 min. As shown in Figure 7a, the surface morphology of the PSi changed, forming pore structures with an average size of approximately 1 μm and an irregular shape. Unlike the mesh pore structure of the PSi samples etched for 10 and 20 min, the diameter of the PSi was large (Figure 7b). However, similar to the PSi sample etched for 30 min, the surface of the PSi sample etched for 40 min did not exhibit fluffy protrusions. Elemental analysis indicated that the PSi sample etched for 40 min contained 7.9 wt% Au, which was higher than those of the PSi samples etched for 10 and 30 min. However, the catalytic effect of the PSi sample etched for 40 min did not exceed those of the PSi samples etched for 10 and 20 min. One possible reason for PNA being subject to a catalytic effect is Au content. In this case, the surface morphology of the Au may have also influenced the catalytic effect. Figure 7d depicts the EDS mapping results of Au for the PSi sample etched for 40 min and reveals a highly uneven distribution of Au and the presence of aggregation. These findings indirectly confirm our hypothesis that the structure and distribution of Au on porous surfaces influence the catalytic effect of PSi on PNA.

**Figure 8:**
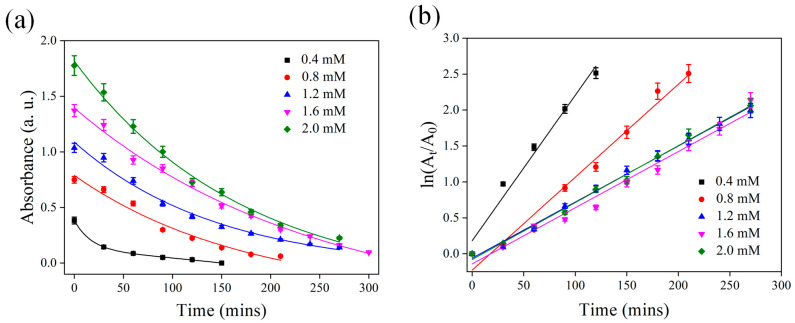
(**a**) Peak absorption (380 nm) change and (**b**) calculated absorption change with PSi etched for 20 min in the presence of different concentrations of PNA.

As a final step, PSi was etched for 20 min, and PNA was catalyzed at different concentrations. This testing method clarified the catalytic effect of PSi on different concentrations of PNA and thereby validated our experimental results. Because we used the same PSi for catalysis, our results confirmed that the produced sample played a role in repeated catalysis. The experimental results are presented in Figure 8. When the PNA concentration was low, the catalytic effect was relatively high. Therefore, we calculated the catalytic rate *k* by using the obtained PNA absorption spectrum change (Table 1). The results indicated that the *k* value was large when the concentration was low. However, when the PNA concentration exceeded 1.2 mM, the catalytic rate of PSi remained unchanged. Therefore, this concentration may be the catalysis and saturation concentration of PSi for PNA. However, by analyzing the catalytic usage times at the same concentration, we discovered that the catalytic efficiency decreased by approximately 35%. Hence, further studies are required to improve the catalytic efficiency and optimize the run time of the catalytic process by adjusting the concentration of auric acid.

## 4. Conclusions

To our knowledge, this is the first study to investigate the use of PSi fabricated by MACE as a carrier for Au NPs and to evaluate its catalytic performance in terms of nitroaromatic compound reduction. Overall, the PSi substrate provided a high surface area and tunable pore size, which facilitated the deposition and catalytic reaction of Au NPs. The Au NPs were then characterized using SEM and EDS, and their catalytic activity was evaluated with nitroaromatic reduction used as a model reaction. The results indicate that Au NPs on PSi exhibit excellent catalytic activity and that the catalytic activity of PSi substrates is affected by the etching time. In addition, a longer etching time results in a larger surface area of PSi and a higher atomic weight percentage of immobilized Au NPs, which leads to higher catalytic activity. However, an excessive etching time results in the aggregation of Au NPs and reduces catalytic activity. These findings have major implications for the development of efficient and cost-effective catalysts for different organic transformation reactions.

## Figures and Tables

**Table 1:** Calculated time constants for PSi etched for 20 min in each cycle of the catalytic process.

Cycles	PNA Concentration (mM)	Time Constant (min^−1^)	Fitting Error (%)
1	1.2	0.01210	2
2	0.4	0.02024	3
3	0.8	0.01294	5
4	1.2	0.00780	4
5	1.6	0.00782	5
6	2.0	0.00791	6

## Data Availability

Not applicable.
